# Supplementary material for: Rationale for a Combination Therapy with the STAT5 Inhibitor AC-4-130 and the MCL1 Inhibitor S63845 in the Treatment of FLT3-Mutated or TET2-Mutated Acute Myeloid Leukemia
Source: Int J Mol Sci. 2021 Jul 28;22(15):8092. doi: 10.3390/ijms22158092 (PMC8347059; doi:10.3390/ijms22158092)
Supplement: Supplementary file 1 [file ijms-22-08092-s001.zip › ijms-1258713-supplementary.pdf]

Supplementary Figures:

Rationale for a combination therapy with the STAT5 inhibitor AC-4-130 and the MCL1 inhibitor S63845 in the treatment of FLT3 mutated or TET2 mutated acute myeloid leukemia

Katja Seipel, Carolyn Graber, Laura Flückiger, Ulrike Bacher and Thomas Pabst

Figure S1.

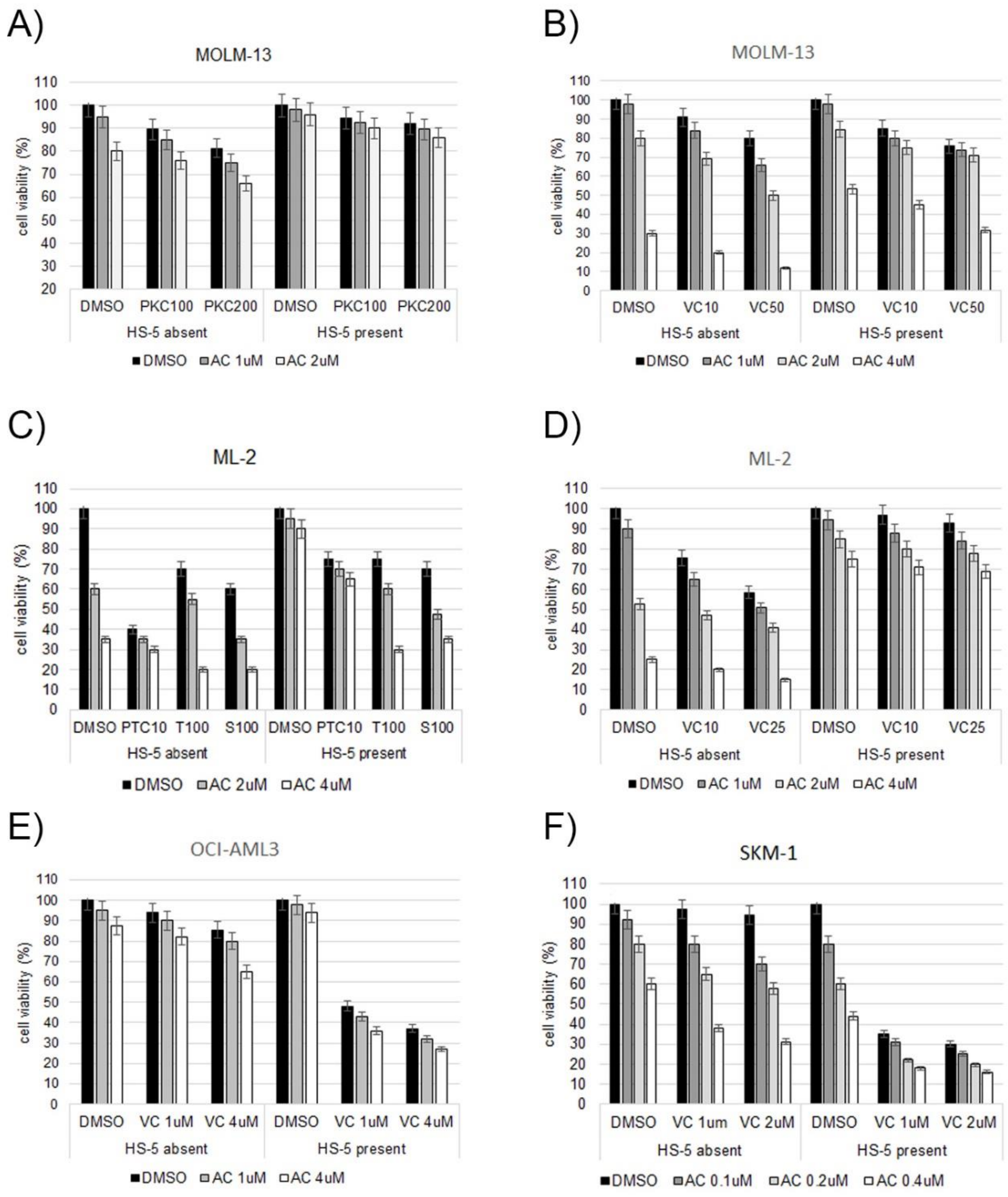

Figure S1. Susceptibility of AML cell lines to various treatment combinations. MOLM-13 (A, B), ML-2 (C, D) and OCI-AML3 (E) and SKM-1 (F) were treated for 20hrs with single compounds and in combination with AC-4-130 (AC) and midostaurin (PKC412), venetoclax (VC), trametinib (T) or S63845 (S). Cell viability was determined in AML cells grown in the absence or presence of HS-5 stroma. Concentrations of inhibitors were nM, except where indicated as uM for venetoclax and AC-4-130. All values are in reference to mock treated cells (= 100% viability).

Figure S2.

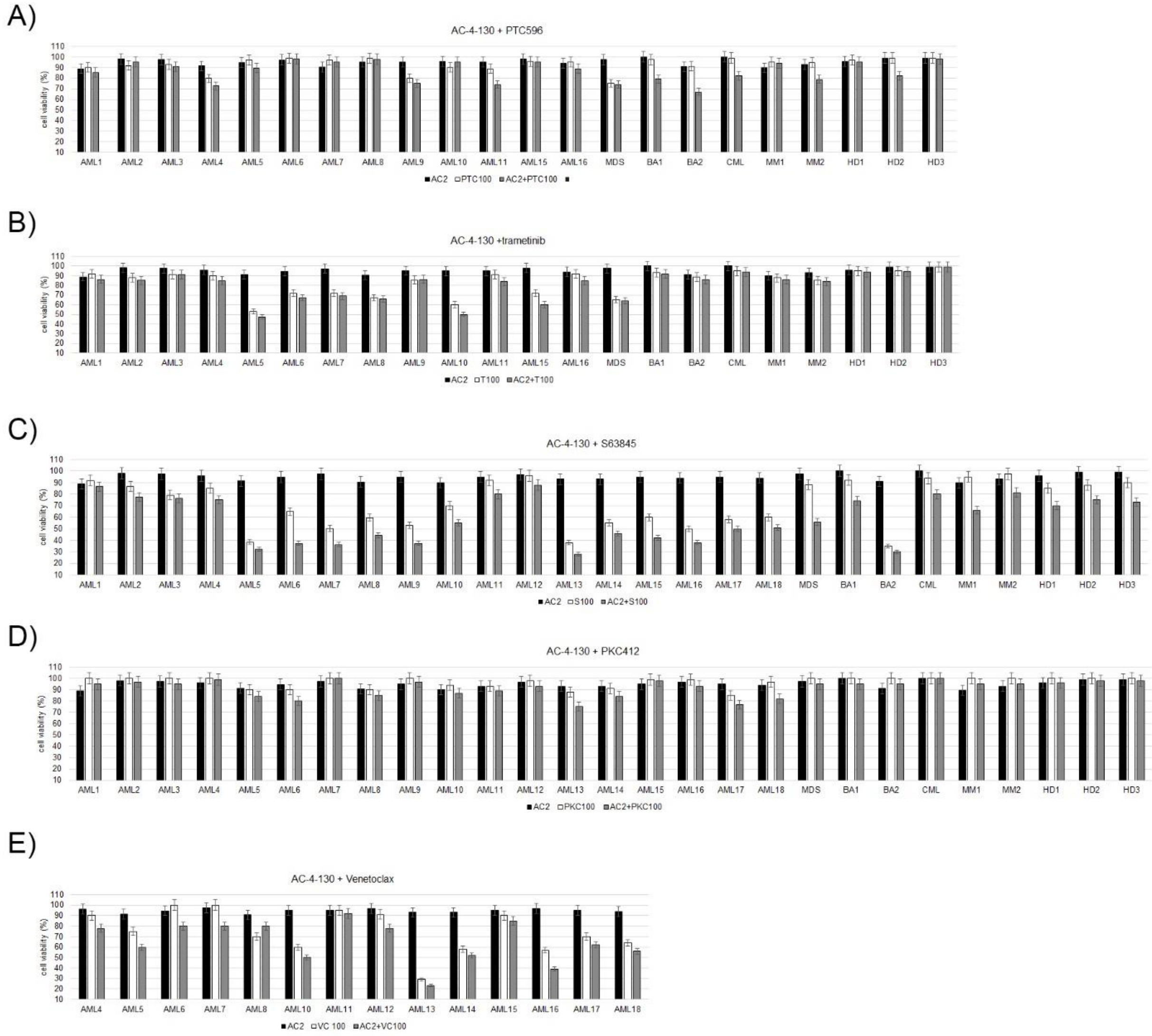

Figure S2. Susceptibility of hematological cells in vitro to various treatment combinations. Cell viability was determined in hematological cells after 20 hour treatment with single compounds and in combination with AC-4-130 and PTC596 (A), AC-4-130 and trametinib (B), AC-4-130 and S63845 (C), AC-4-130 and midostaurin (D), AC-4-130 and venetoclax (E). AML, acute myeloid leukemia; ALL, acute lymphocytic leukemia; CML, chronic myeloid leukemia; HD, healthy donor; MDS, myelodysplastic syndrome; MM, multiple myeloma. For the FLT3 gene the mutant allele ratio, for the TP53 gene the mutant allele frequency (VAF) are indicated in parentheses.
